# Synchronizing Protein Traffic to the Primary Cilium

**DOI:** 10.3389/fgene.2019.00163

**Published:** 2019-03-08

**Authors:** Wladislaw Stroukov, Axel Rösch, Carsten Schwan, Abris Jeney, Winfried Römer, Roland Thuenauer

**Affiliations:** ^1^Signalling Research Centres BIOSS and CIBSS, University of Freiburg, Freiburg, Germany; ^2^Faculty of Chemistry and Pharmacy, University of Freiburg, Freiburg, Germany; ^3^Medical Faculty, Institute of Experimental and Clinical Pharmacology and Toxicology, University of Freiburg, Freiburg, Germany; ^4^Faculty of Biology, University of Freiburg, Freiburg, Germany

**Keywords:** primary cilium, epithelial cells, protein trafficking, somatostatin receptor 3, nephrocystin-3

## Abstract

The primary cilium is able to maintain a specific protein composition, which is critical for its function as a signaling organelle. Here we introduce a system to synchronize biosynthetic trafficking of ciliary proteins that is based on conditional aggregation domains (CADs). This approach enables to create a wave of ciliary proteins that are transported together, which opens novel avenues for visualizing and studying ciliary import mechanisms. By using somatostatin receptor 3 (SSTR3) as model protein we studied intracellular transport and ciliary import with high temporal and spatial resolution in epithelial Madin-Darby canine kidney (MDCK) cells. This yielded the interesting discovery that SSTR3, besides being transported to the primary cilium, is also targeted to the basolateral plasma membrane. In addition, we found a similar behavior for another ciliary protein, nephrocystin-3 (NPHP3), thus suggesting a potential correlation between ciliary and basolateral trafficking. Furthermore, our CAD-based system allowed assembling a large dataset in which apical and basolateral surface SSTR3 signals could be compared to ciliary SSTR3 signals on a single cell level. This enabled to generate novel complementary evidence for the previously proposed lateral import mechanism of SSTR3 into the cilium along the plasma membrane.

## Introduction

The primary cilium is a cellular organelle that maintains a particular composition of proteins and lipids, although the ciliary membrane and the surrounding plasma membrane are continuous (Singla and Reiter, 2006). The capacity of the cilium to concentrate or exclude specific proteins is of critical importance for its function as a signaling organelle. Various ciliopathies, such as Joubert syndrome and nephronophthisis, show an altered concentration of signaling proteins at the primary cilium (Shi et al., 2017). The key structure controlling access to the cilium is a “ciliary gate” that is located at the base of the cilium. A presumable part of the ciliary gate is a membrane diffusion barrier that can hinder free diffusion of specific membrane proteins (Vieira et al., 2006; Hu and Nelson, 2011; Hu et al., 2012; Reales et al., 2015); thus assisting in the distinctive concentration of proteins at the cilium. However, alternative mechanisms that do not necessarily require a diffusion barrier have also been described: membrane proteins can be excluded from the cilium by anchoring to the actin cytoskeleton (Francis et al., 2011), and, *vice versa*, ciliary proteins could be selectively anchored within the cilium (Hu and Nelson, 2011). In addition, the anterograde intraflagellar transport machinery could actively drag proteins into the cilium, whereas proteins leaking from the cilium could be rapidly endocytosed (Hu and Nelson, 2011). To distinguish between these different mechanisms, which might all be at work for different classes of ciliary proteins, novel methods are required that enable to directly image and quantify the biosynthetic transport of ciliary proteins toward the cilium and into the cilium.

Here, we applied an approach based on conditional aggregation domains (CADs) (Rivera et al., 2000; Thuenauer et al., 2014) to synchronize biosynthetic trafficking of ciliary proteins. The particular strength of synchronization is that it ensures that a cohort of a protein of interest is transported together and reaches the primary cilium only along biosynthetic trafficking routes. Hence, the synchronized movement of proteins makes it easier to distinguish and follow individual transport steps. This cannot be achieved with other techniques, such as ciliary fluorescence recovery after bleaching (FRAP), in which the cilium can be refilled with newly synthesized proteins and also with proteins recruited from recycling compartments. In addition, due to the basic limits of optical resolution, it is difficult to selectively bleach the primary cilium but not adjacent regions such as the periciliary membrane or subapical vesicles.

To demonstrate the capabilities of our approach we applied the CAD-based system to study ciliary trafficking in epithelial Madin-Darby canine kidney (MDCK) cells. MDCK cells form their primary cilium from a midbody remnant and therefore do not exhibit a ciliary pocket (Bernabe-Rubio et al., 2016). It is not well-understood if and how the presence of a ciliary pocket influences ciliary trafficking, since most studies on ciliary trafficking had been carried out in cells bearing a ciliary pocket. We analyzed the intracellular and ciliary trafficking of the ciliary model protein somatostatin receptor 3 (SSTR3) utilizing a CAD-tagged synchronizable version of SSTR3. SSTR3 has been reported to be expressed in kidney epithelial cells (Bates et al., 2003) and a recent study has identified a mechanism for ciliary import of SSTR3 via lateral transport along the periciliary membrane regulated by tubby-family proteins and the intraflagellar transport sub-complex A (IFT-A) (Badgandi et al., 2017). Surprisingly, our approach revealed that SSTR3 is also transported to the basolateral plasma membrane of MDCK cells. In addition, we found a similar behavior for another ciliary protein, nephrocystin-3 (NPHP3), thus suggesting a potential link between ciliary trafficking and basolateral trafficking. Finally, by analyzing the correlation between apical SSTR3 and ciliary SSTR3, and between basolateral SSTR3 and ciliary SSTR3, on a single cell level, we provide quantitative evidence for lateral import of SSTR3 from the apical plasma membrane into the ciliary membrane also in MDCK cells, which are devoid of a ciliary pocket.

## Results and Discussion

### Design of a Synchronizable Version of SSTR3

SSTR3 is a rhodopsin-family G protein-coupled heptahelical receptor that is expressed in many tissues, including the nervous system and kidneys (Bates et al., 2003). It is imported into the primary cilium, presumably by binding the IFT-A complex in a manner coordinated by the tubby-family proteins TULP3 and TUB (Badgandi et al., 2017).

**Figure 1:**
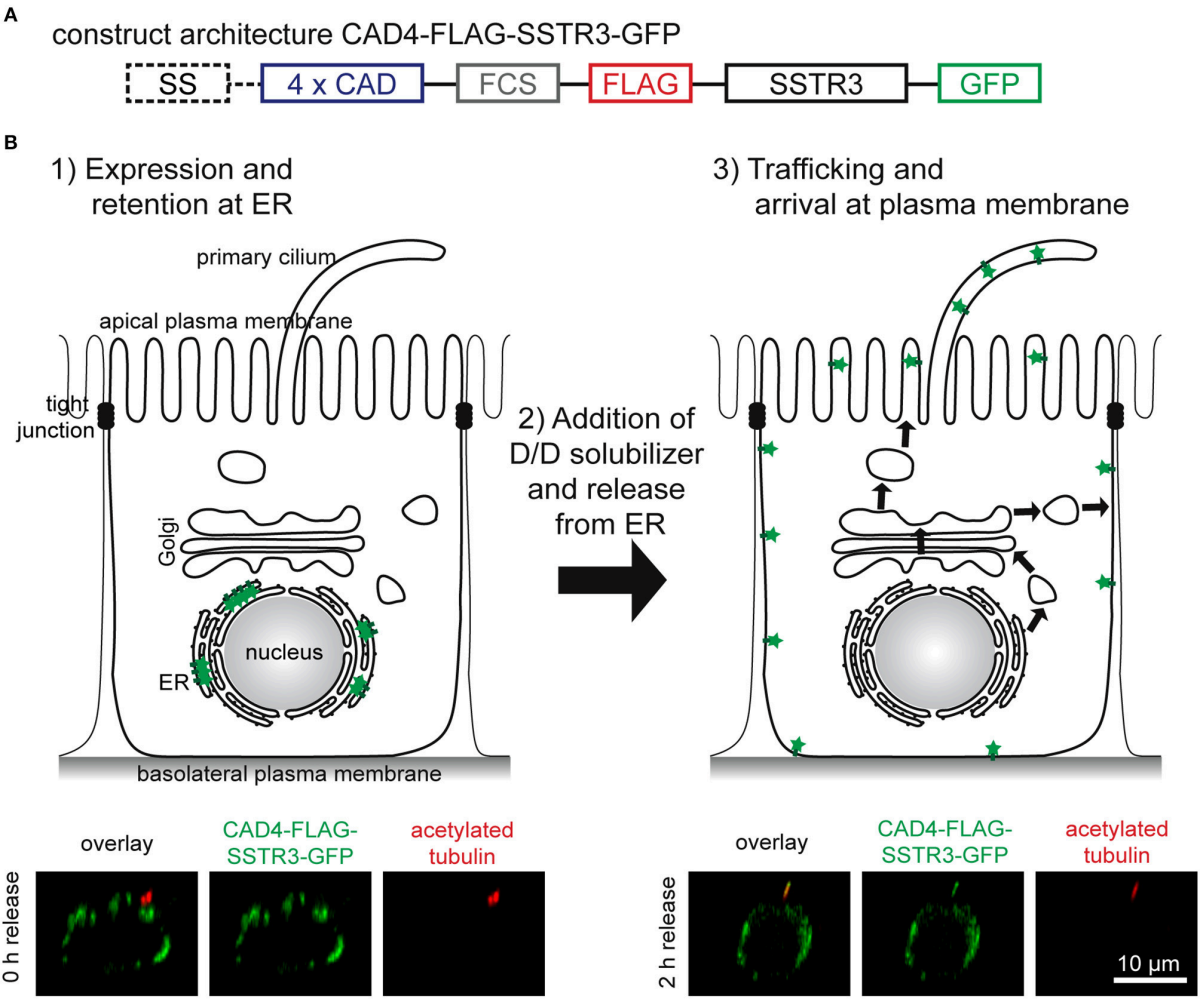
Functional principle of CAD-mediated synchronized protein trafficking to the primary cilium of epithelial cells. **(A)** Architecture of the construct CAD4-FLAG-SSTR3-GFP. A signal sequence (SS) derived from the human growth hormone ensures insertion into the ER membrane during synthesis. This is followed by four copies of conditional aggregation domains (4 X CAD), a furin cleavage site (FCS) ensuring removal of the CAD-domains by furin in the trans-Golgi network, a FLAG-tag, SSTR3, and GFP. **(B)** After transient expression, the resulting proteins CAD4-FLAG-SSTR3-GFP are retained at the ER due to CAD-mediated formation of aggregates (1). The aggregates can be rapidly dissolved by addition of D/D-solubilizer (2). This generates a wave of proteins synchronously transported to the plasma membrane (3). Whereas, the upper images show schematic representations, the lower images display apico-basal cross-sections of representative cells expressing CAD4-FLAG-SSTR3-GFP (green) and cilia highlighted by anti-acetylated tubulin staining (red) derived from confocal image stacks.

Motivated by the successful synchronization of another rhodopsin-family receptor, rhodopsin (Thuenauer et al., 2014), we reapplied the tagging strategy for generating a synchronizable version of SSTR3. The architecture of the resulting construct CAD4-FLAG-SSTR3-GFP is shown in Figure 1A. A signal sequence (SS) derived from the human growth hormone ensures insertion of the protein of interest into the endoplasmic reticulum (ER) membrane during synthesis. Four copies of CAD domains (4 X CAD) are responsible for synchronization: After expression, large CAD-mediated aggregates are formed (Figure 1B, step 1), which are retained at the ER. After addition of the membrane permeable small molecule D/D-solubilizer, the aggregates are dissolved, and a synchronous wave of the protein of interest is released from the ER (Figure 1B, step 2) and then trafficked to the plasma membrane (Figure 1B, step 3). The CAD domains are separated from the remaining construct by a furin cleavage site (FCS) enabling the trans-Golgi network (TGN)-resident protease furin to remove the CADs at the TGN (Thuenauer et al., 2014). In addition, a FLAG-tag is linked to the extracellular N-terminus of SSTR3 and thereby accessible at the plasma membrane from the extracellular medium, whereas a green fluorescent protein (GFP) is attached at the cytoplasmic C-terminus of SSTR3. In the lower row of Figure 1B it can be seen that the hybrid protein CAD4-FLAG-SSTR3-GFP formed intracellular clusters before release (0 h), but reached the primary cilium after 2 h of release. Interestingly, also a presumable basolateral localization of FLAG-SSTR3-GFP became evident after 2 h of release.

Taken together, the construct CAD4-FLAG-SSTR3-GFP is successfully retained at the ER after expression and is trafficked to the primary cilium after ER release upon addition of D/D-solubilizer.

### Optimization of Transfection

**Figure 2:**
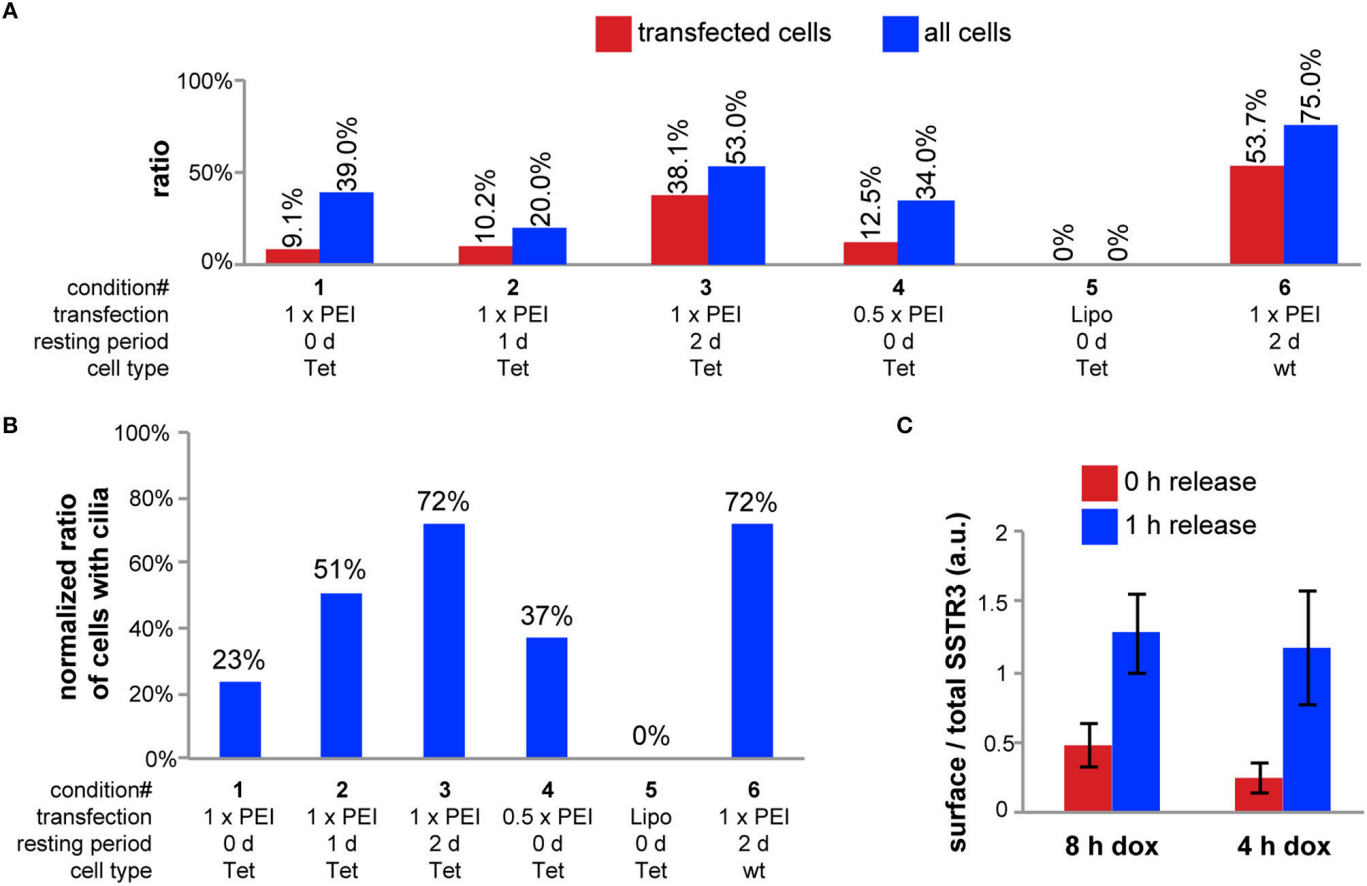
Optimization of the transfection and induction. **(A)** For conditions 1-3, tet-MDCK cells grown on transwell filters were transfected using 1.5 μg pTet-CAD4-FLAG-SSTR3-GFP and 5 μl PEI stock solution for 12 h. For condition 4, only the half amount of DNA and PEI was used. For condition 5, tet-MDCK cells were transfected using 1.6 μg pTet-CAD4-FLAG-SSTR3-GFP and 4 μl Lipofectamine 2000. For condition 6, wt MDCK cells were transfected using 0.75 μg of pTet-CAD4-FLAG-SSTR3-GFP and 0.75 μg of pWHE644 (encoding a tet transactivator) and 5 μl PEI stock solution. After transfection, all samples were washed with medium and incubated at 37°C for the indicated resting periods. Then, in all samples CAD4-FLAG-SSTR3-GFP expression was induced by treatment with doxycycline for 8 h. To characterize cilia expression, cells were fixed, stained for α-tubulin, and imaged with a confocal microscope. The ratio of SSTR3-expressing cells and of all cells showing cilia (red and blue bars, respectively) were manually counted from n > 3 randomly chosen regions with lateral dimensions of 200 × 200 μm. **(B)** Ratio of CAD4-FLAG-SSTR3-GFP-transfected cells displaying cilia normalized to the ratio of all cells displaying cilia for the same conditions as in **(A)**. **(C)** Transwell filter grown wt MDCK cells were transfected using 0.75 μg of pTet-CAD4-FLAG-SSTR3-GFP and 0.75 μg of pWHE644 and 5 μl PEI stock solution for 12 h and then allowed to rest for 2 d. CAD4-FLAG-SSTR3-GFP expression was induced by treatment with doxycycline for 8 or 4 h. Next, D/D-solubilizer was either added for 1 h (1 h release) or not (0 h release) and FLAG-SSTR3-GFP that had arrived at the cell surface was stained by apical and basolateral addition of anti-FLAG antibodies. After fixation, cells were imaged with a confocal microscope. To assess the leakiness of the ER retention of CAD4-FLAG-SSTR3-GFP, the ratio of total surface signals from anti-FLAG staining and GFP signals representing the total CAD4-FLAG-SSTR3-GFP amount were determined for *n* > 25 single cells per condition and averaged.

In initial tests we used a plasmid encoding for CAD4-FLAG-SSTR3-GFP under the control of a constitutive CMV-promoter (pCMV-CAD4-FLAG-SSTR3-GFP). Transient transfection experiments with this plasmid showed that the major prerequisite for unbiased quantification of individual cells can be achieved: only single isolated cells within a polarized, transwell filter-grown MDCK monolayer were transfected using PEI as transfection reagent for 12 h (data not shown). However, we recognized that transfected cells rarely displayed cilia, which presumably resulted from cellular stress during transient transfection. Therefore, we made the plasmid pTet-CAD4-FLAG-SSTR3-GFP, for expressing the same construct under control of a tetracycline (tet)-inducible promoter, with the goal to separate the time point of transfection from the time point of the onset of expression. After establishing a cell line stably expressing a tet transactivator (tet-MDCK), we used the plasmid pTet-CAD4-FLAG-SSTR3-GFP to optimize conditions for best cilia expression (Figure 2A). Increasing the resting period between transfection with pTet-CAD4-FLAG-SSTR3-GFP and induction of CAD4-FLAG-SSTR3-GFP expression with doxycycline (dox) from 0 d to 2 d increased the ratio of transfected cells bearing cilia from 9.1 to 38.1%, respectively. Reducing the PEI transfection dose by a factor of two only slightly improved the cilia expression ratio from 9.1 to 12.5%, respectively, for 0 d resting periods. Using Lipofectamine 2000 as transfection reagent completely failed and abolished virtually all cilia. Thus, using PEI as transfection reagent followed by a 2 d resting period appeared to be the best option.

However, the ratio of transfected cells expressing cilia was still low. To identify the cause, we compared cilia expression rates for untransfected tet-MDCK cells and wt MDCK cells, which revealed that more wt MDCK cells expressed cilia: 75% of polarized wt MDCK cells had cilia, compared to only 53% of polarized tet-MDCK cells. Hence, to further optimize CAD4-FLAG-SSTR3-GFP expression conditions, we chose an alternative strategy based on wt MDCK cells, which we co-transfected with a plasmid encoding a tet transactivator (pWHE644) and pTet-CAD4-FLAG-SSTR3-GFP. After 2 d of rest before dox induction, 53.7% of transfected cells expressed cilia as compared to 75% of all cells in the sample. To further analyze the contributions from resting periods vs. cell/clone type, we calculated to which extend cilia expression was lowered in transfected cells in comparison to all cells for the investigated conditions (Figure 2B). This verified again the positive effect of an increase of the resting period. Importantly, transfection lowered cilia expression in both, tet-MDCK and wt MDCK cells, to the same extend (reduction to 72%), which indicates that the parameters “resting period” and “cell/clone type” independently contribute to the cilia expression ratio.

Previous work with CAD-synchronized proteins in our group revealed that different proteins display varying degrees of leakiness during the period of ER retention (unpublished). Therefore, we sought to minimize the duration of dox-induced protein synthesis to lower leakiness from the ER. For quantification, we stained cell surface FLAG-SSTR3-GFP with apically and basolaterally applied FLAG-antibodies and then measured the SSTR3 cell surface to total SSTR3 signal ratios in cells induced for 8 or 4 h with dox and treated with D/D-solubilizer for 0 or 1 h (Figure 2C). This showed that induction for 4 h yields sufficient expression levels but results in lower leakiness. With these optimized conditions we verified by Western blot analysis that furin cleavage of CAD4-FLAG-SSTR3-GFP occurred (Supplementary Figure 1).

In summary, a dox-inducible strategy allowed for a 2 d resting period after transfection, which drastically increased the ratio of transfected cells expressing cilia. In addition, 4 h of dox induction resulted in sufficient expression levels together with a low ER leakiness of the construct CAD4-FLAG-SSTR3-GFP.

### Quantification of Polarized Cell Surface and Ciliary Arrival

**Figure 3:**
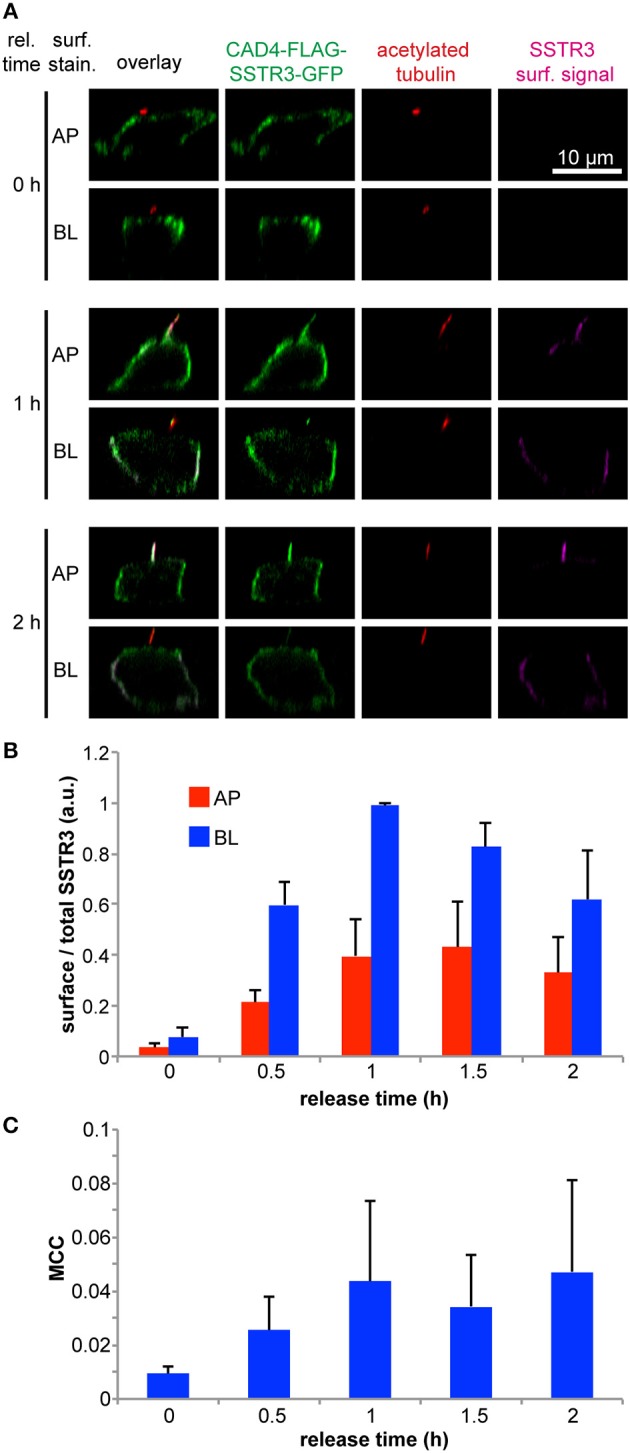
Time-resolved arrival of SSTR3 at the apical and basolateral plasma membrane and at the primary cilium. **(A)** Transwell filter grown wt MDCK cells were transfected using 0.75 μg of pTet-CAD4-FLAG-SSTR3-GFP and 0.75 μg of pWHE644 and 5 μl PEI stock solution for 12 h and then allowed to rest for 2 d. CAD4-FLAG-SSTR3-GFP (green) expression was induced by treatment with doxycycline for 4 h followed by treatment with D/D-solubilizer to induce ER release for the indicated times. Afterwards FLAG-SSTR3-GFP that had arrived at the apical (AP) or basolateral (BL) cell surface (magenta) was stained using anti-FLAG antibodies administered only to apical or basolateral side, respectively. After fixation, the primary cilium was stained using anti-acetylated tubulin antibodies (red). **(B)** Quantification of FLAG-SSTR3-GFP apical (AP) and basolateral (BL) cell surface arrival after the indicated ER release periods. The mean values from three independent experiments are shown. In one experiment, the surface to total FLAG-SSTR3-GFP signal ratios were determined from *n* > 50 individual cells per condition, averaged, and normalized to the maximum value of the time series before the mean values for the repeated experiments were calculated. **(C)** Manders' co-localization coefficient (MCC) between FLAG-SSTR3-GFP and acetylated tubulin as ciliary marker in dependence of the CAD4-FLAG-SSTR3-GFP release time. The mean values from three independent experiments with each *n* > 30 cells per condition are displayed.

As released FLAG-SSTR3-GFP was found not only at the primary cilium, but also at the basolateral plasma membrane, we sought to further investigate its trafficking to the apical and basolateral cell surface in a time-dependent manner (Figures 3A,B). This revealed that FLAG-SSTR3-GFP arrived at the basolateral plasma membrane at an ~2-fold higher amount than at the apical plasma membrane throughout the tracked 2 h release period (Figure 3B). (Note: We use the term “apical plasma membrane” in this context to denominate the combination of the ciliary membrane and non-ciliary apical plasma membrane).

In addition, we found for another ciliary protein, NPHP3, that it was also partially targeted to the basolateral plasma membrane after ER release (Supplementary Figure 2). Interestingly, NPHP3 uses a different mechanism for ciliary import than SSTR3: NPHP3 can be anchored to the cytosolic leaflet of the plasma membrane via a myristoyl lipid anchor. This anchor can be masked by UNC119, thus creating a cytosolic complex and enabling import into the cilium via a cytosolic route. In the primary cilium UNC119 is removed with the help of the small GTPase ARL3 and NPHP3 can insert its lipid anchor into the ciliary membrane (Wright et al., 2011; Jaiswal et al., 2016). Our data suggests that basolateral localization in epithelial cells could be a feature of many ciliary proteins, and indeed, basolateral localization has been observed before for e.g., Smoothened (Corbit et al., 2005) and polycystin-1 (Roitbak et al., 2004; Su et al., 2015). These observations indicate a startling link between basolateral and ciliary trafficking. To further substantiate this hypothesis, we investigated the localization of the activated Smoothened mutant SmoA1 (Boehlke et al., 2010) during establishment of polarity in MDCK cells (Supplementary Figure 3). This revealed that SmoA1 remained intracellular in non-polarized MDCK cells. Only after polarization SmoA1 localized to the primary cilium and also to the basolateral plasma membrane. This indicates that polarity is essential for SmoA1 plasma membrane localization and that ciliary and basolateral localization seem to be established within the same timeframe.

One explanation for the coincidence between ciliary and basolateral localization could lie in the convergence or overlap between ciliary and basolateral trafficking mechanisms. For example, the small GTPase Rab8 and the clathrin adaptor AP-1 have been implicated in both, ciliary and basolateral membrane trafficking (Kaplan et al., 2010; Carvajal-Gonzalez et al., 2012; Rodriguez-Boulan and Macara, 2014). Another possibility could be the presence of a transcytotic delivery pathway from the basolateral plasma membrane to the primary cilium. This would be consistent with our observation (Figure 3B) that the arrival of SSTR3 at the basolateral plasma membrane peaked at 1 h, whereas the arrival at the apical plasma membrane peaked slightly later, at 1.5 h. To directly assess the existence of transcytotic delivery of SSTR3 we carried out an experiment in which we first released CAD4-FLAG-SSTR3-GFP for 1 h and then applied primary anti-FLAG antibodies to the basolateral medium to label FLAG-SSTR3-GFP at the basolateral surface. After further 1 or 4 h of incubation at 37°C, we probed for transcytosis with apically applied secondary antibodies recognizing the primary anti-FLAG antibodies. This showed that indeed transcytosis of SSTR3 occurred (Supplementary Figure 4). Therefore, we suggest that after ER release SSTR3 is trafficked to the apical and basolateral plasma membrane, and concomitantly transcytosis from the basolateral to the apical side takes place.

An open question is the physiological role of the basolateral pool of ciliary proteins. Is this pool capable of transducing signals to a similar extent as the ciliary pool? Or is it acting as a reservoir that can be rapidly recruited to the primary cilium if required? It will be highly interesting to investigate these hypotheses in future and our synchronization system offers a powerful experimental handle to approach these questions.

We further aimed to directly visualize and quantify the arrival of FLAG-SSTR3-GFP at the primary cilium. To this end we analyzed the co-localization of FLAG-SSTR3-GFP with the ciliary marker acetylated tubulin via a Manders' co-localization coefficient (MCC) in dependence of the ER release time (Figure 3C). Our analysis revealed that this approach requires several measures to quantify ciliary co-localization of FLAG-SSTR3-GFP correctly: (1) the threshold in the acetylated tubulin channel needs to be carefully set so that only signals directly from the primary cilium remain and other acetylated tubulin signals from within the cells are effectively cut off. (2) A co-localization analysis is inherently limited by the resolution of the used microscope. To minimize artifacts that derive from limited resolution, we only analyzed the MCC for cells that had clearly protruding cilia and not from cells where e.g., cilia were sharply tilted and were therefore localized in the same image plane as the surrounding apical plasma membrane.

Over the course of 2 h, we could observe an increase of FLAG-SSTR3-GFP in the primary cilium of MDCK cells, reaching saturation after approximately 1–1.5 h (Figure 3C). Since the MCC is defined as the sum of co-localizing signal normalized to the total signal (see equation (1) in section Quantification of Polarized Cell Surface Arrival and Ciliary Arrival), its value represents the fraction of SSTR3 localized in the primary cilium. It peaks around 0.05, meaning that 5% of the total SSTR3 protein is present in the primary cilium. This supports an active accumulation of SSTR3 in the primary cilium, because the ciliary volume is smaller by factor of 2,700–6,800 than the total cellular volume (Nachury, 2014) and hence random delivery of SSTR3 into the primary cilium could only account for MCC values of <0.0004.

**Figure 4:**
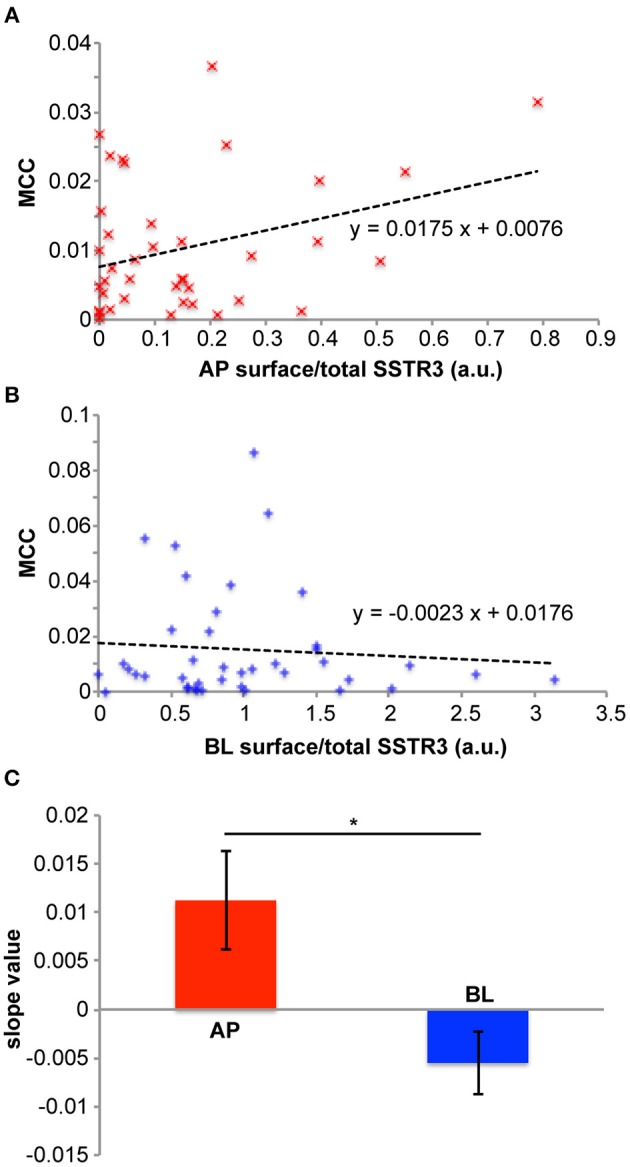
Relations between SSTR3 ciliary arrival and SSTR3 apical and basolateral surface localization. **(A–C)** cells were treated as described in Figure 3. **(A)** depicts a representative diagram showing how the ciliary FLAG-SSTR3-GFP localization (measured by the Manders' co-localization coefficient (MCC) between FLAG-SSTR3-GFP and acetylated tubulin as ciliary marker) depends on apical (AP) FLAG-SSTR3-GFP (measured by the ratio of the apical surface FLAG-SSTR3-GFP staining signal to the total FLAG-SSTR3-GFP signal) in individual cells after 1 h of ER release (red ×). A linear fit to the data is displayed as black dashed line, together with the corresponding equation. **(B)** Same as in **(A)**, but for basolateral (BL) FLAG-SSTR3-GFP. **(C)** The slope values for linear fits of ciliary FLAG-SSTR3-GFP vs. apical (AP) or basolateral (BL) FLAG-SSTR3-GFP were averaged from three independent experiments. Statistical analysis was carried out with a two-tailed paired *t*-test, and ^*^ indicates *p* < 0.05.

A further strength of our system is that we are able to analyze ciliary arrival in populations of individual cells. To illustrate this, we plotted the distribution of MCC values vs. the normalized apical FLAG-SSTR3-GFP surface signal (Figure 4A) and vs. the normalized basolateral FLAG-SSTR3-GFP surface signal (Figure 4B) for individual cells after 1 h of release. First, no saturation effects were observable for cells with higher apical or basolateral surface signals, which indicates that we operated with expression levels that were low enough to not exhaust the transport machinery toward or into the primary cilium. Second, there exists a positive correlation between MCC and the normalized apical SSTR3 surface signal, i.e., in cells with higher apical signals also higher signals were found in the cilium. In contrast, the MCC and the normalized basolateral SSTR3 surface signal exhibited a negative correlation. For quantification, a linear fit to the data of Figures 4A,B was carried out and the slope of the resulting line was defined as measure for the correlation between MCC and surface signal. Analysis of three independent replicates revealed a significant difference between the slope for the apical side and the slope for the basolateral side (Figure 4C), with the apical slope always assuming positive values. This is consistent with the assumption that SSTR3 was imported into the primary cilium via lateral transport along the apical plasma membrane in MDCK cells, which corresponds to the proposed tubby-family and IFT-A-mediated mechanism for SSTR3 ciliary import (Badgandi et al., 2017). Since the tubby/IFT-A-mediated mechanism was established using cells bearing a ciliary pocket, our results suggest that in cells that do not possess a ciliary pocket, such as MDCK cells (Bernabe-Rubio et al., 2016), the same mechanism for SSTR3 ciliary import is employed.

Taken together, we showed that our CAD-based synchronization approach enables to quantify polarized cell surface arrival and ciliary import of proteins in a time-dependent manner on a single cell and population level. Furthermore, the synchronization system was instrumental for uncovering a potential link between ciliary and basolateral trafficking.

### Live-Cell Imaging of Ciliary Targeting

**Figure 5:**
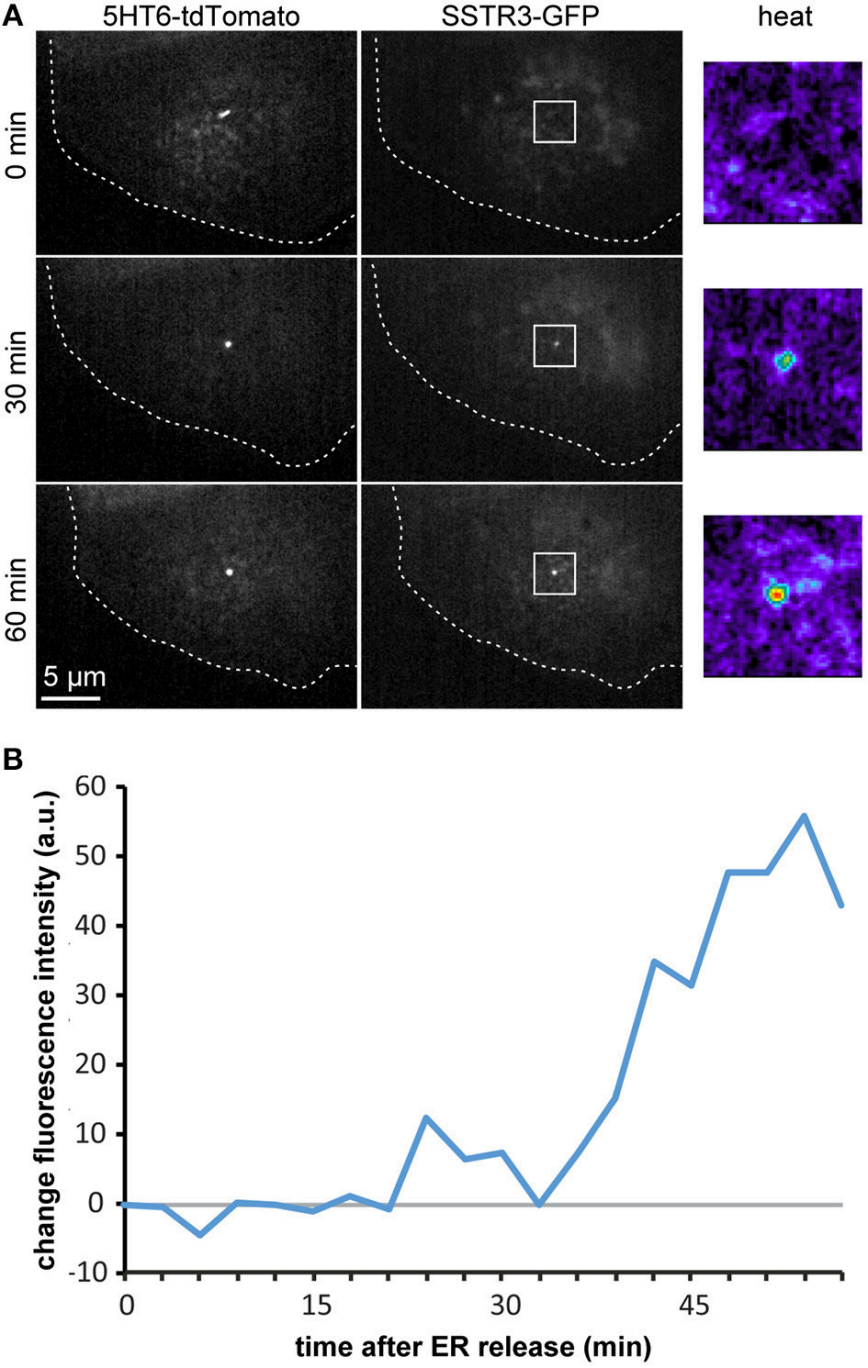
Live-cell imaging and quantification of ciliary arrival. **(A)** Polarized MDCK cells were transfected with pCMV-CAD4-SSTR3-GFP and p5HT6-tdTomato as ciliary marker. 4 h after transfection, CAD4-SSTR3-GFP was released from the ER by addition of D/D-solubilizer (*t* = 0 min). Time lapse images from a single confocal image plane at the level of the primary cilium are displayed and increase of SSTR3-GFP fluorescence in selected areas (white boxes) is also shown in false-color magnifications (heat). **(B)** The cilium outlined by the signal from the 5HT6-tdTomato channel was used to mask the SSTR3-GFP channel in order to quantify the increase of SSTR3-GFP fluorescence intensity at the cilium in dependence of the time after addition of D/D-solubilizer.

To demonstrate the feasibility of monitoring transport into the primary cilium in live cells, we co-transfected cells with pCMV-CAD4-SSTR3-GFP and p5HT6-tdTomato as a ciliary marker (Berbari et al., 2008; Lesiak et al., 2018). As it can be seen in Figure 5 and in Supplementary Movie 1, this enabled to image the gradual accumulation of SSTR3-GFP at the primary cilium by spinning disc confocal microscopy. To quantify the imaging data (Figure 5A), we measured the signal of SSTR3-GFP co-localizing with 5HT6-tdTomato over time (Figure 5B). The kinetics determined by the live cell imaging approach largely corresponded to the kinetics that was determined by the averaged values from multiple fixed single cells (Figure 3C), with an onset of ciliary localization at 30 min that increased until ~1 h after release.

In summary, these experiments show that our system is capable to visualize and quantify the ciliary arrival of proteins in live cells.

## Conclusions

Here we introduce an approach to synchronize the biosynthetic trafficking of ciliary proteins based on tagging with CADs. We show that this system is broadly applicable for proteins imported into the primary cilium by different mechanisms, as illustrated for SSTR3, which is imported via lateral transport along the plasma membrane, and NPHP3, which is imported via the cytosol in a state where its membrane anchor is masked. By using SSTR3 as model protein, we demonstrated that creating a synchronous wave of proteins targeted to the primary cilium is a fruitful approach to uncover mechanistic details of ciliary delivery. In particular, CAD-based synchronization facilitates direct observation of intracellular transport to the primary cilium starting from a state in which the cilium is free from the protein of interest. This allows a selective investigation of biosynthetic delivery to the primary cilium, which is not possible with other methods such as ciliary FRAP. In addition, many individual cells can be monitored in parallel and we present a framework to quantify the kinetics of polarized cell surface arrival and ciliary arrival in epithelial cells. This framework laid the foundation for the discovery that ciliary proteins, including SSTR3 and NPHP3, can exhibit also basolateral localization in epithelial cells. In future, the synchronization system will be instrumental to study the relation between basolateral and ciliary trafficking mechanisms, including the characterization of a potential transcytotic route from the basolateral plasma membrane to the primary cilium. Furthermore, the data we generated allowed to investigate the correlation between apical SSTR3 and ciliary SSTR3 and between basolateral SSTR3 and ciliary SSTR3 on a single cell level. This yielded complementary quantitative evidence for lateral import of SSTR3 into the cilium along the plasma membrane. Finally, we demonstrated that CAD-based synchronization also allowed imaging and quantifying ciliary import in live cells, thus opening novel possibilities to monitor ciliary trafficking in real time with high resolution in time and space.

## Materials and Methods

### Cell Culture and Protein Expression

MDCK strain II cells (wt MDCK) were a gift from Enrique Rodriguez-Boulan (Weill Cornell Medical College, NY, USA) and were maintained in Dulbecco's modified Eagle's medium (DMEM) supplemented with 5% fetal calf serum (FCS) at 37°C and 5% CO_2_ in 10 cm culture dishes and passaged every 2–3 d.

For growing polarized monolayers, cells were cultured on transwell filters (#3401 from Corning-Costar) for 4 d. Transfections were carried out using polyethylenimine (PEI; linear, 25 kDa; Polysciences, stock concentration 1 μg/μl) or Lipofectamine 2000 (Thermo Fisher Scientific) using the dosages indicated in the main text. Doxycycline (Sigma Aldrich) was used at a concentration of 5 μg/ml and D/D-solubilizer (Clontech/Takara) at a concentration of 5 μM.

For generating the cell line stably expressing a tet-regulated transactivator, wt MDCK cells were transfected with the plasmid pWHE644 linearized with *Ahd I* (Herr et al., 2011) and afterwards clones were selected with puromycin (Sigma Aldrich). Six clones were selected and tested using a plasmid encoding for GFP under control of a tet-promoter. Two clones with the tightest repression and highest inducibility after treatment with doxycycline for 12 h were selected. They were further inspected for their capability to form well-polarized monolayers by culturing the cells on transwell filters for 4 d followed by immunofluorescence staining with antibodies directed against the basolateral marker β-catenin (ab32572 from Abcam), the apical marker podocalyxin (produced by the hybridoma cell line R26.4C obtained from the Developmental Studies Hybridoma Bank at the University of Iowa), and against α-tubulin to visualize cilia (T5168 from Sigma Aldrich) and the best clone was chosen for further experiments (tet-MDCK).

### Plasmids

The plasmids pCMV-CAD4-FLAG-SSTR3-GFP, pTet-CAD4-FLAG-SSTR3-GFP, and pCMV-CAD4-SSTR3-GFP were cloned using Gibson assembly with a vector encoding pCMV-FM4-GFP (Thuenauer et al., 2014) as backbone and using the murine SSTR3 sequence from ptdTomato-SSTR3-N-17 (gift from Michael Davidson, Addgene plasmid # 58132). pWHE644 was obtained from TET Systems GmbH (Heidelberg, Germany). The plasmid p5HT6-tdTomato was cloned from pEGFPN3-5HT6 (gift from Kirk Mykytyn, Addgene plasmid # 35624).

### Surface Staining and Immunofluorescence

Staining for CAD4-FLAG-SSTR3-GFP arrived at the cell surface was carried out with anti- DYKDDDDK/FLAG antibodies (637301 from BioLegend) that were diluted in medium 1:200 and applied to the apical or basolateral side of transwell filter-grown cells for 15 min at room temperature. After two washes with phosphate buffered saline (PBS), cells were fixed with a 4% formaldehyde solution for 15 min at room temperature.

For immunofluorescence (IF), cells were permeabilized with SAPO medium (PBS supplemented with 0.2% bovine serum albumin and 0.02% saponin) for 30 min at room temperature. Next secondary anti-rat Alexa647-tagged antibodies (A21247 from Thermo Fisher Scientific) directed against the primary anti-FLAG antibodies were applied for 1 h, followed by staining with anti-acetylated tubulin antibodies (T7451 from Sigma Aldrich) for 1 h, and then secondary anti-mouse Cy3-tagged antibodies (715-166-1500 from Jackson ImmunoResearch) directed against the anti-acetylated tubulin antibodies for 30 min. After excising filters with a scalpel, they were mounted in DABCO-medium (Müller et al., 2017).

### Quantification of Polarized Cell Surface Arrival and Ciliary Arrival

Cells were imaged with a confocal microscope (Nikon A1R) equipped with a 60X oil immersion objective with a numerical aperture (N.A.) of 1.49 and lasers emitting at 405, 488, 561, and 641 nm. Image stacks covering complete cell heights were recorded and used for quantification with a custom-written Matlab program (Thuenauer et al., 2014; Müller et al., 2017). To ensure proper quantification at a single cell level, only cells that were surrounded by non-expressing cells were quantified. First, background signal levels were determined from regions covering non-expressing cells and subtracted from the images. For quantifying apical/basolateral cell surface arrival, the ratio of the intensities from apical/basolateral cell surface staining and GFP intensities representing total FLAG-SSTR3-GFP amounts were determined in individual cells and averaged.

Ciliary arrival was quantified via calculating the MCC (Dunn et al., 2011) between the FLAG-SSTR3-GFP signal above background and the signal above background from the ciliary marker acetylated tubulin according to the formula

(1) $$\begin{array}{l} \;MCC=\;\frac{\sum_{i}{SSTR3}_{i}^{coloc}}{\sum_{j}{SSTR3}_{j}} \end{array}$$

where ${SSTR3}_{i}^{coloc}$ is the FLAG-SSTR3-GFP signal from voxels that were also positive for acetylated tubulin, and *SSTR*3_*j*_ is the signal from voxels positive for FLAG-SSTR3-GFP. To minimize potential artifacts resulting from the limited resolution of the confocal microscope, only cells with clearly visible and protruding cilia were measured.

### Live Cell Imaging

Live cell imaging was carried out with a Zeiss Observer Z1 microscope equipped with a 100X N.A. 1.46 oil immersion objective, lasers emitting at 488 and 561 nm, a spinning disc unit (Yokogawa CSU-X1), and a Photometrics Prime sCMOS camera. MDCK cells were grown on glass bottom dishes for 5 d, starved for 48 h and then transfected with pCMV-CAD4-SSTR3-GFP and p5HT6-tdTomato as ciliary marker.

### Statistics

If not indicated otherwise, mean values were determined from three independent experiments. Error bars represent the standard error of the mean. Statistical analysis was carried out with a two-tailed paired *t*-test, and ^*^ indicates *p* < 0.05.

## Data Availability

The raw data supporting the conclusions of this manuscript will be made available by the authors, without undue reservation, to any qualified researcher.

## Author Contributions

WS performed experiments and wrote the manuscript. AR, AJ, and CS performed experiments. WR contributed to study design. RT contributed to study design, developed the methods, performed experiments, and wrote the manuscript. All authors proofread the manuscript.

### Conflict of Interest Statement

The authors declare that the research was conducted in the absence of any commercial or financial relationships that could be construed as a potential conflict of interest.
