# Supplementary material for: Synchronizing Protein Traffic to the Primary Cilium
Source: Front Genet. 2019 Mar 8;10:163. doi: 10.3389/fgene.2019.00163 (PMC6419537; doi:10.3389/fgene.2019.00163)
Supplement: Supplementary file 2 [file Data_Sheet_1.docx]

Supplementary Material

Synchronizing protein traffic to the primary cilium

Wladislaw Stroukov^1,2+^, Axel Rösch^1,2+^, Carsten Schwan^3^, Abris Jeney^1,4^, Winfried Römer^1,4^, Roland Thuenauer^1,4*^

^1^ Signalling Research Centres BIOSS and CIBSS, University of Freiburg, Freiburg, Germany,

^2^ Faculty of Chemistry and Pharmacy, University of Freiburg, Freiburg, Germany

^3^ Institute of Experimental and Clinical Pharmacology and Toxicology, Medical Faculty, University of Freiburg, Freiburg, Germany.

^4^ Faculty of Biology, University of Freiburg, Freiburg, Germany

**^+^ equal contribution**

*** Correspondence:** roland.thuenauer@gmail.com

# Supplementary Methods

**Plasmids**

The plasmid pNPHP3-GFP-CAD4 was constructed by Gibson assembly and encodes for the first 203 amino acids of human NPHP3 (derived from a plasmid provided by Gerd Walz (University Medical Center Freiburg)), GFP, and 4 x CAD domains. Please note that furin cleavage of CAD4 in the NPHP3-GFP-CAD4 construct is not possible due to the cytosolic localization of NPHP3-GFP-CAD4.

**Western Blotting**

Cells were lysed with RIPA buffer (20 mM Tris (pH 8.0), 0.5% (w/vol) Na-deoxycholate, 13.7 mM NaCl, 10% (vol/vol) glycerol, 0.1% (w/vol) SDS, 2 mM EDTA) supplemented with protease and phosphatase inhibitors (200 μM pefabloc, 11 μM leupeptin, 0.8 μM aprotinin, and 1 mM Na-orthovanadate, 1% (vol/vol)). Lysates were centrifuged for 10 min at 4°C and 18000 x g to pellet debris. The protein content of supernatants was measured with a BCA assay kit (Thermo Fisher). 60 µg of protein was loaded per lane and Western blot analysis was performed as described previously (Zheng et al. 2017). CAD4-FLAG-SSTR3-GFP and furin-cleaved FLAG-SSTR3-GFP was detected using anti-GFP antibodies (sc-9996 from Santa Cruz Biotechnology) and actin was detected with anti-actin antibodies (A 5316 from Sigma Aldrich) as loading control.

**Cell lines**

MDCK II cells stably expressing SmoA1-Venus (Boehlke et al. 2010) were a gift from Wolfgang Kühn (University Medical Center Freiburg).

# Supplementary Data

## Supplementary Figures


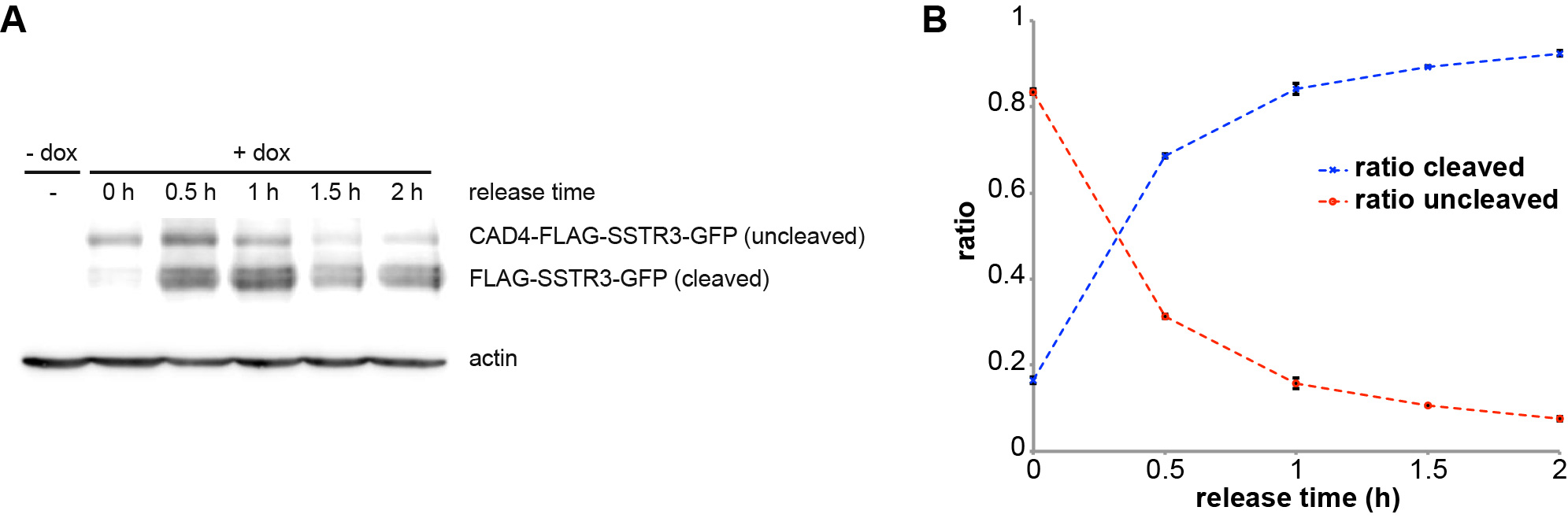
**Supplementary Figure 1: Analysis of furin cleavage for the construct CAD4-FLAG-SSTR3-GFP.** Polarized wt MDCK cells were transfected using 0.75 µg of pTet-CAD4-FLAG-SSTR3-GFP and 0.75 µg of pWHE644 and 5 µl PEI stock solution for 12 h and then allowed to rest for 2 d. CAD4-FLAG-SSTR3-GFP expression was induced by treatment with doxycycline for 4 h (+dox) or not (-dox), followed by treatment with D/D-solubilizer to induce ER release for the indicated times. After lysis with with RIPA buffer, samples were subjected to Western blot analysis. (A) Representative Western blot result. (B) Quantification of furin cleavage. The ratio of cleaved proteins was calculated from the intensity of the cleaved band divided by the sum of the intensities of the cleaved and the uncleaved band for each time point. For comparison, the ratio of uncleaved proteins is also displayed, which corresponds to 1 - ratio of cleaved proteins. The graph shows the mean values from three independent experiments, error bars represent the standard error of the mean.


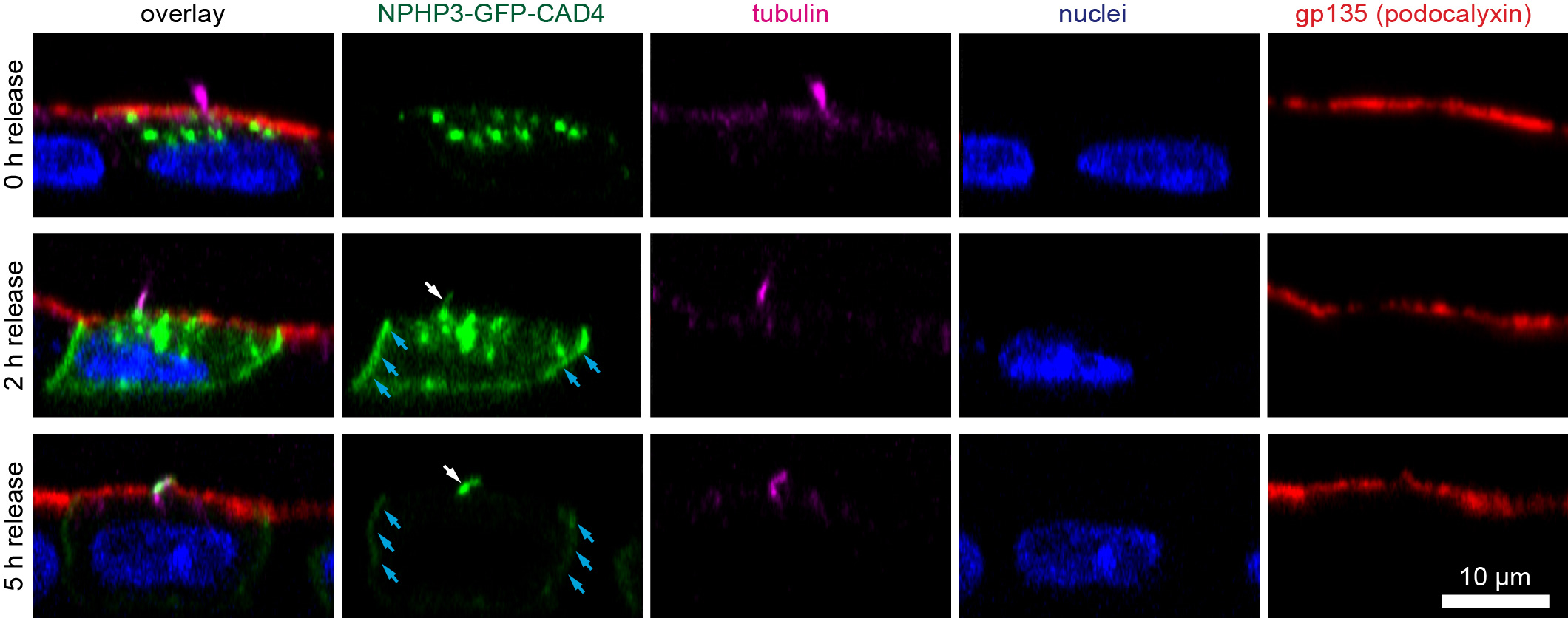


**Supplementary Figure 2: A synchronizable version of NPHP3.** Polarized filter-grown MDCK cells were transfected with NPHP3-GFP-CAD4 (green) and incubated with D/D-solubilizer to induce ER release for the indicated times. After fixation, cells were stained against tubulin (magenta) to visualize cilia, the apical marker gp135/podocalyxin (red), and nuclei were stained with DAPI (blue). Apico-basal cross-sections of representative cells derived from confocal image stacks are displayed. White arrows point to ciliary NPHP3-GFP, whereas cyan arrows point to basolateral NPHP3.


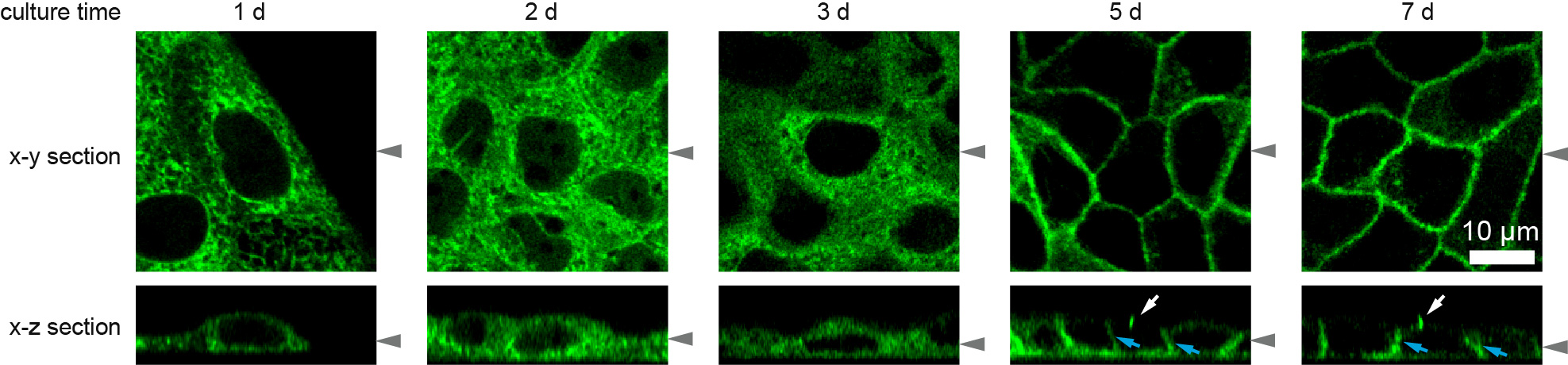


**Supplementary Figure 3: Localization of SmoA1 in polarizing MDCK cells.** MDCK cells stably expressing SmoA1-Venus (green) were seeded on glass bottom dishes and live cell imaging with a confocal microscope was carried out at the indicated time points. In the upper row x-y sections extracted from confocal image stacks are depicted, and the lower row shows the corresponding x-z sections. Gray arrowheads in the upper row indicate the positions of the x-z sections, and gray arrowheads in the lower row indicate the positions of the x-y sections. White arrows point to ciliary SmoA1-Venus, whereas cyan arrows point to basolateral SmoA1-Venus.


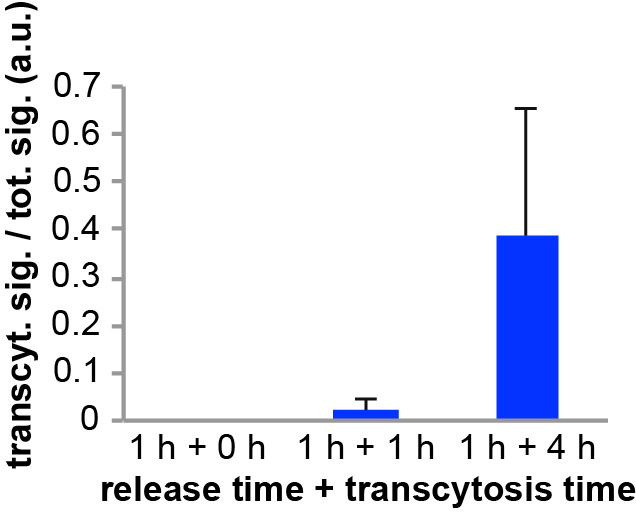
**Supplementary Figure 4: Analysis of SSTR3 transcytosis.** Polarized transwell filter grown wt MDCK cells were transfected using 0.75 µg of pTet-CAD4-FLAG-SSTR3-GFP and 0.75 µg of pWHE644 and 5 µl PEI stock solution for 12 h and then allowed to rest for 2 d. CAD4-FLAG-SSTR3-GFP expression was induced by treatment with doxycycline for 4 h, followed by treatment with D/D-solubilizer for 1 h (release time). To probe for transcytosis, primary anti-FLAG antibodies were applied to the basolateral side to label basolateral FLAG-SSTR3-GFP. After 0 h, 1 h, or 4 h at 37°C (transcytosis time), transcytosed anti-FLAG antibody-FLAG-SSTR3-GFP complexes were labeled by apical application of secondary Alexa647-tagged antibodies directed against the primary anti-FLAG antibodies. To relatively quantify transcytosis, signals from the Alexa647 channel (indicating transcytosed SSTR3) were normalized to total FLAG-SSTR3-GFP signals for individual cells. The averaged transcytosis signal/total signal values for n > 8 cells per condition are displayed, error bars represent the standard error of the mean.

## Supplementary Movies

**Supplementary Movie 1: Live cell recording of SSTR3-GFP ciliary import.** MDCK cells were co-transfected with the ciliary marker 5HT6-tdTomato (grayscale fluorescence image top left) and CAD4-SSTR3-GFP (grayscale fluorescence data top right, heat-scaled data bottom right, and magnification of the heat-scaled data bottom left) and CAD4-SSTR3-GFP was released from the ER by addition of D/D-solubilizer (t = 0 min).

# Supplementary References

Boehlke, Christopher, Mikhail Bashkurov, Andrea Buescher, Theda Krick, Anne-Katharina John, Roland Nitschke, Gerd Walz, and E Wolfgang Kuehn. 2010. “Differential Role of Rab Proteins in Ciliary Trafficking: Rab23 Regulates Smoothened Levels.” *Journal of Cell Science* 123 (Pt 9): 1460–67. https://doi.org/10.1242/jcs.058883.

Zheng, Shuangshuang, Thorsten Eierhoff, Sahaja Aigal, Annette Brandel, Roland Thuenauer, Sophie de Bentzmann, Anne Imberty, and Winfried Römer. 2017. “The Pseudomonas Aeruginosa Lectin LecA Triggers Host Cell Signalling by Glycosphingolipid-Dependent Phosphorylation of the Adaptor Protein CrkII.” *Biochimica et Biophysica Acta (BBA)-Molecular Cell Research* 1864 (7): 1236–45. https://doi.org/https://doi.org/10.1016/j.bbamcr.2017.04.005.
